# Methods for Assessing Long-Term Exposures to Outdoor Air Pollutants

**DOI:** 10.1007/s40572-017-0169-5

**Published:** 2017-10-24

**Authors:** Gerard Hoek

**Affiliations:** Institute for Risk Assessment Sciences (IRAS), PO Box 80178, 3508 TD Utrecht, Netherlands

**Keywords:** Outdoor air pollution, Exposure, Fine particle, Model, Satellite

## Abstract

**Purpose of Review:**

Epidemiological studies of health effects of long-term exposure to outdoor air pollution rely on different exposure assessment methods. This review discusses widely used methods with a special focus on new developments.

**Recent Findings:**

New data and study designs have been applied, including satellite measurements of fine particles and nitrogen dioxide (NO_2_). The methods to apply satellite data for epidemiological studies are improving rapidly and have already contributed significantly to national-, continental- and global-scale models. Spatiotemporal models have been developed allowing more detailed temporal resolution compared to spatial models. The development of hybrid models combining dispersion models, satellite observations, land use and surface monitoring has improved models substantially. Mobile monitoring designs to develop models for long-term UFP exposure have been conducted.

**Summary:**

Methods to assess long-term exposure to outdoor air pollution have improved significantly over the past decade. Application of satellite data and mobile monitoring designs is promising new methods.

## Introduction

Assessment of long-term exposure to ambient air pollution for epidemiological studies remains challenging. Early cohort studies characterized exposure to individual participants by assigning the average concentration measured at one or a few central sites within a city to each participant from this city [1, 2]. These cohort studies thus limited the health effect analysis to between-city exposure contrast. However, many studies have documented significant variation of outdoor air pollution at a small scale within urban areas for important pollutants such as nitrogen dioxide (NO_2)_, black carbon and ultrafine particles [3–5]. Within-city spatial contrasts may be even larger than the between-city contrast for particularly combustion-related pollutants [4]. An analysis in the Women’s Health Initiative study suggested that the risks of fine particles related to within-city contrasts were larger than for between-city contrasts [6]. To characterize the intra-urban contrasts, approaches beyond direct monitoring were applied and further developed, including exposure indicator variables (e.g. traffic intensity at a residential address or distance to a major road), interpolation methods (e.g. kriging, inverse distance weighing), dispersion models and land use regression models [7]. Recent trends in epidemiology studies of long-term air pollution exposures have led to further development of these models.

One recent development in air pollution epidemiology is the use of very large populations (populations of over 1 million adults) to be able to estimate air pollution effects in the low-exposure range. For this, often, national cohorts based upon administrative databases [8–10] are used. These studies have to rely on existing monitoring data, as targeted campaigns would be too costly. Routine surface air pollution-monitoring networks often do not cover the full population, thereby limiting assessment of exposure to part of the population [10]. To overcome these limitations, air pollution monitoring from satellites is increasingly applied.

Another new development is the necessity to study health effects of long-term exposure to particle metrics beyond the regulated PM_2.5_ and PM_10_, including ultrafine particles (UFPs), black carbon (BC) or other combustion-related air pollutants. Routine monitoring data is typically limited to regulated pollutants and does not measure ultrafine particles and black carbon. Hence, there is much less information on health effects of long-term exposure for these components than for fine particles, despite the potentially high health risks of these combustion-related particles [5, 11]. UFP and BC are characterized by high temporal and spatial variabilities [5, 11]. Models for these highly variable pollutants require a higher spatial resolution than what is achievable with routine monitoring data. In recent years, mobile and short-term monitoring campaigns have been conducted to develop land use regression models for ultrafine particles and black carbon.

There is increased recognition of the limitations of dispersion/chemical transport modelling, land use regression modelling and monitoring to assess individual exposure for epidemiological studies [7]. As an example, routine monitoring data is typically spatially sparse and temporally rich, whereas typical land use regression models are spatially more detailed but temporally less rich. Studies have started to combine different methods such as land use or chemical transport modelling and monitoring data using a variety of approaches, exploiting the strengths of different methods.

In this review, first, an overview of methods will be presented. The review will focus on new developments particularly regarding mobile monitoring and the use of satellite-based remote sensing. Second, examples of studies that have combined different methods will be discussed. We complete the review with a discussion of issues that apply to all methods, including the assessment of historical exposure contrasts and potential bias related to residence-based exposure assessment. The text on land use regression (LUR) models builds on a previously published book chapter [12]. An exhaustive review of all studies that have contributed to long-term air pollution exposure assessment methodology is not possible, because of the large volume of studies. Rather, examples are presented from different research approaches.

## Overview of Methods

Table 1 presents an overview of methods that have been applied in epidemiological studies of long-term exposure to outdoor air pollution. An extensive discussion of the principle of most methods has been published previously [7, 12, 13].

**Table 1:** Methods to assess long-term average outdoor air pollution exposure for epidemiological studies

Methods	Principle	Applications in epidemiological studies	Comment
Monitoring	Measured value from surface-monitoring stations directly assigned to participants	[1, 2, 6, 106]	Nearest station (within a certain distance) or average of all stations in a city
Interpolation	Assign interpolations of measured values from monitoring stations, using ordinary kriging, inverse distance weighing or other geo-statistical methods	[17, 18]	Applied for ozone and PM_2.5_, pollutants with limited local variation
Satellite monitoring	Surface PM_2.5_ and NO_2_ concentrations obtained by combining measured column concentration and vertical distribution of a chemical transport model (CTM)	[10, 69–71, 84•]	Combines remote sensing and CTM for vertical gradient; often supplemented with additional land use and traffic data
Indicators of exposure	Traffic intensity nearest to the road, distance to a major road	[92, 107]	Not a quantitative pollution estimate
Land use regression modelling	Fixed site and more recently mobile monitoring to develop empirical models using traffic, population and land use predictor variables	[9, 84•, 92]	Spatial and spatiotemporal models; increase in predictor variables such as satellite and dispersion/chemical transport models
Dispersion/chemical transport modelling	Modelling of dispersion of emission from source to receptors using deterministic models	[9, 55–58]	Recently more on a fine spatial scale

Not in order of assumed correctness, but in detail of spatial variation

### Monitoring and Interpolation

Monitoring data from typically one or a few (routine) monitors per city have been assigned to individual subjects by calculating a city average and assigning this average to all subjects in the city [2]. Another simple approach is to assign monitoring data from the nearest monitor to individual subjects [14].

The merits of direct assignment of measured concentrations from typically a routine monitoring network include low cost, consistency of monitoring methods and often a long period of monitoring. The use of direct monitoring data further avoids potential problems related to the use of models with limited or uncertain validity. A main limitation is the lack of characterization of intra-urban contrasts related to motorized traffic emissions and other local sources, because networks are typically not spatially dense enough.

Simple assignment of a city average measured concentration may be defensible if the variability between cities is large, such as in the American Cancer Society (ACS) study covering much of the USA [2]. Further rationales for this approach include the following: (1) More detailed modeling of outdoor concentrations at, for example, residential addresses, without data on time activity of participants may add additional noise to exposure assessment. The assumption that subjects spend a large fraction of time in their city of residence is less strong than the assumption that they spend all time at their residence. (2) For components that are difficult to model because emission factors are not available and specific sources are not known, using direct monitoring may be attractive. This argument has been made in a recent LUR study of the oxidative potential of particles [15]. While LUR models were developed with good explanatory power, the model was likely not specific for oxidative potential. (3) Assigning a city average concentration to all subjects in a city likely leads to a Berkson error in exposure, which does not lead to biased risk estimates when applied in an epidemiological study [16].

Interpolation of monitoring data has been used to overcome some of the limitations of assignment of a city average or data from the nearest station [7]. Methods that have been used include inverse distance weighing [17] and kriging [18]. Interpolation methods use data from multiple stations and assign more weight to stations that are at a shorter distance from the receptor point (e.g. residential address). Although interpolation methods provide a spatially more resolved pattern than a simple average, the assumed smooth spatial change of concentration may be too simple in urban areas. In urban areas, local sources such as major roads may result in sharper changes than assumed in interpolation methods. Interpolation is more attractive for components with a relatively small local source component, including, for example, PM_2.5_, and in more rural areas.

A recent study in Oakland, CA, used a mobile monitoring approach to map air pollution across the city [19]. A large number of repeats for all road segments in the study area allowed the direct use of the average measured concentration by road segment as an estimate of exposure in epidemiological studies without the need for model development [19]. This approach could be applied in epidemiological studies when relatively small neighborhoods are studied. The use of mobile monitoring for model development is discussed in more detail below.

Developments of low-cost sensors may allow more spatially dense networks in the future [20, 21]. It is unlikely that networks will be sufficiently dense to use them directly for exposure assessment. Larkin and Hystad discuss the use of (low-cost) sensors in individual exposure assessment [22].

### Land Use Regression Models

Land use regression models are empirical (regression) models derived by combining monitoring of air pollution at a limited number of locations and collection of (land use) variables via geographic information systems (GIS), which can potentially predict the measured spatial variation [12, 13]. Typically, (linear) regression models are developed using a variety of methodologies to select predictors, such as supervised stepwise procedures or machine-learning algorithms. The regression model is then applied to a large number of locations in the study area where no measurements are available to predict the point-specific air pollutant concentration, e.g. the residential, school or work addresses of subjects, in an epidemiological study. LUR modeling uses as predictor variables various traffic representations, population/address density, land use and variables such as altitude. Land use regression is an empirical approach in contrast to dispersion/chemical transport models which are based upon physical principles and actual emission data. This explains the difficulty to transfer LUR models from one study area to another. Epidemiologists have applied land use regression because the models are directly based upon locally measured air pollution data, the relative ease of applying the method, the lower demand on input data compared to dispersion/chemical transport models and the typically good performance of the models (high explained variance of measured spatial variation).

Land use regression modeling has been based upon existing routine monitoring as well as on specially designed monitoring campaigns. Routine monitoring networks have often been used for national or continental models. These large-scale national or continental LUR models are limited to routinely measured pollutants (typically no novel or emerging air pollution measures such as UFP or oxidative potential). Depending on the representation of sites in the network, they may not cover local traffic impacts sufficiently, as is the case in North American models which include only very few traffic-monitoring sites [23]. The latter concern applies less to European networks, as these typically include a sizable fraction of traffic-monitoring stations [24•].

Next to LURs based on routine monitoring, LURs have been developed in the framework of dedicated studies. In these studies, specially designed measurement networks operating for a limited time are typically spatially denser within urban areas than routine networks and the distribution of site types (e.g. traffic, background) and pollutants can be optimized for the study objectives. The disadvantages of purpose-designed monitoring include the additional cost and the limited temporal coverage of the measurements. The typical purpose-designed monitoring campaign consists of between 7–14-day sampling campaigns that are repeated between one and four times in 1 year to capture all seasons. The method has been applied to the gaseous components NO_2_, NO_*x*_ and VOCs and to particulate matter such as PM_2.5_, PM_10_ and soot. Measurements are typically not conducted simultaneously at all sites and thus require a reference site to adjust for temporal variation.

### New Developments in LUR Modeling

Recently, LUR models for particle composition have been published, responding to one of the key needs in air pollution epidemiology, which is the identification of the hazardous components of the complex air pollution mixture [25, 26•, 27]. Compared to NO_2_ and PM_2.5_, these models are based upon fewer routine monitoring sites. In a North American study, models developed for elemental carbon (EC), organic carbon (OC), S and Si for six US cities explained between-city variability well, but within-city variability was not well explained [26•]. In a European study based on dedicated sampling, within-study area models for Fe, Cu and Zn had good explanatory power, whereas models for the other five elements (S, Si, V, Ni and K) had moderate to low explanatory powers [25]. This was attributed to the lack of specific land use predictors for these latter components, affected especially by non-traffic sources (Fe, Cu and Zn reflect non-tailpipe traffic emissions). Models for PAH and levoglucosan (emitted by wood smoke) also had moderate explanatory power, related to the difficulty to assess the spatial distribution of wood smoke emissions [28, 29].

Recently, UFP LUR models based on fixed-site monitoring approach have been reported in the cities of Amsterdam, Augsburg and Rome and a Swiss area [30–33]. A limitation of these studies is the modest number of sites, which could be monitored with this approach. An advantage compared to the later discussed mobile monitoring campaigns is the higher precision of the site average, leading to typically higher model *R* ^2^.

Methodologically, progress has been made in assessing the robustness of LUR models in relation to the number of monitoring sites [34, 35]. The ability of models to predict variability of measured concentrations at independent sites not used for modeling especially increased from models that used only 20 monitors to those that used about 80 in two studies conducted in the city of Girona, Spain, [35] and the Netherlands [34]. Beyond 80 sites, only modest improvements in explained variance were observed.

Different methods have been applied for variable selection in the development of LUR models, including supervised stepwise procedures and the deletion-substitution algorithm (DSA) [36]. DSA is a machine-learning algorithm that uses a covariate search algorithm to fit a generalized linear model, minimizing the cross-validation mean squared error [36]. Reid and co-workers applied a variety of machine-learning methods to develop models to predict PM_2.5_ concentrations related to forest fires [37]. Few comparisons have been made between the performance of different variable selection methods. In a study in Girona, Spain, performance measured by hold-out validation statistics did not differ substantially between DSA and the supervised stepwise method used in the European Study of Cohorts for Air Pollution Effects (ESCAPE) study [35]. In a Canadian mobile monitoring study, no material difference was found in performance between stepwise selection and a machine-learning method [38]. Based upon internal cross-validation statistics, random forest (a machine-learning method) performed better than supervised stepwise selection to develop prediction models [39].

One limitation of many LURs is their lack of temporal variation. To overcome this limitation, spatiotemporal models were developed which account for the common unbalanced spatiotemporal data structure by incorporating spatial and temporal predictors in one model [27, 40]. Spatiotemporal models for monthly average fine and coarse particle concentrations were developed for the USA, combining spatial and temporal predictors in a generalized additive modelling framework [41, 42]. Models for fine particles had higher performance than models for coarse particles. Spatiotemporal models for daily averages have been developed using satellite data; see the “Satellite Remote Sensing” section. Spatiotemporal models offer the investigator more flexibility in defining biologically relevant exposure metrics than the spatial models based upon an annual average. An example is exposure estimation in birth cohort studies where trimester-specific estimates are frequently used to assess health effects [43].

### LUR Based Upon Short-Term or Mobile Monitoring Campaigns

A further new development is the modeling of particle metrics such as UFP or BC, characterized by highly variable spatial and temporal distributions, with a large number of short-term measurements or mobile monitoring campaigns [38, 44–47]. The instruments available to measure ultrafine particle or total particle number concentrations are typically too expensive or require too much operator interference to be used in the monitoring campaigns based upon fixed sites where instruments are left unattended for 1–2 weeks. Instead, short-term campaigns with a large number of repeated measurements using only one or few instruments can be conducted. These short-term campaigns can be differentiated into short-term fixed-site monitoring and on-road mobile monitoring. Short-term fixed site monitoring typically uses one or few instruments at a large number of locations with short sampling periods per location (15–60 min) and a small number of repeats at each site. In a study in Girona, Spain, over 600 sites were measured for 15-min periods each, outside rush hours in the summer [45]. In later studies, three repeats of 30-min averages in different seasons were performed [48, 49] or a single repeat of 60-min measurements [46]. On-road mobile monitoring has also been used with typically even shorter sampling periods in a specific street but with more repeats. Mobile campaigns are typically conducted with one instrument while driving a car or bicycle or walking [44, 47, 50, 51•]. For model development, the mobile data are often aggregated per street segment. GIS predictors are collected for the centroid of the street segment.

Because of the short duration per site, the potential for temporal bias is even larger than in the design with 1–2-week samples at fixed sites. One or more (urban or rural background) reference sites are often used to adjust for temporal variation. The strength of the design is the large number of sites that can be measured, allowing inclusion of a variety of site types that takes into account the complexity of urban areas, e.g. different traffic intensities and configurations of streets. Site or route selection is less demanding than site selection for longer-term sampling, where safety is an important requirement. The design is further efficient in terms of personnel cost [45, 51•].

Because of the short sampling period, temporal fluctuations affect the site average typically used for modelling more than in studies using longer sampling times. Therefore, these short-term and mobile sampling campaigns are less precise in determining spatial variation of long-term average concentrations, unless a large number of repeats are performed. This was shown in a recent mobile monitoring study in Oakland, CA, involving a car equipped with state-of-the-art air pollution monitors driving a large number of routes [19]. This study documented that up to about 10 repeated driving days, the stability of the measured average increased, and after about 10–20 driving days, the stability of the average did not improve appreciably with further repeats [19].

Despite the often limited precision of the site average concentrations, robust land use regression models have been developed because of the large number of sites. LUR models based upon mobile and short-term campaigns typically have moderate explained variation of the short-term measurements (model *R* ^2^ less than 50%). The moderate model *R* ^2^ values are a result of the low precision of the site averages due to the short measurement periods [48, 51•]. The model *R* ^2^ may substantially underestimate the explained variance of the model for spatial variability of average concentrations based upon longer averaging times per site. Two studies documented that the models explained a larger fraction of the variation in external measurements based upon longer averaging times per site than the model *R* ^2^ itself [48, 49]. A Dutch model for BC based upon monitoring at 160 sites three times for 30 min per site had a model *R* ^2^ of 35%, but explained 61% of the variation of a proxy for BC measured during three 14-day average campaigns in the Netherlands [48]. The explanation for the observation of a low model *R* ^2^ and robust spatial model is that the measurement error in a continuous dependent variable does not lead to bias in linear regression analysis [48]. Mobile campaigns with a large number of repeats per road segment have typically also reported larger model *R* ^2^ values of up to 80% [38, 52, 53].

A concern with the application of models based upon on-road mobile monitoring is that they may overestimate exposures at residential addresses, even when the influence of high-emission vehicles in front of the monitoring vehicle is excluded in the analysis. A recent Dutch study documented that models based upon mobile monitoring using an electric car predicted about 30% higher UFP exposure than short-term fixed-site monitoring models in the same study [51•]. However, predictions of the two models were highly correlated, suggesting that the application in epidemiological studies would lead to similar conclusions. Further work is needed to assess whether this finding also applied in settings where mobile monitoring campaigns included freeways, as the distance to homes is much larger.

The application of UFP LUR models based on mobile monitoring in epidemiological studies of health effects of long-term exposure to UFP has just started [54]. Further development of the method is useful, particularly with respect to validation with external data with longer averaging times per site. Most mobile models have been developed within single metropolitan areas. There is a need to develop large-scale models to match the study area of (national) cohorts.

### Dispersion/Chemical Transport Modelling

Dispersion/chemical transport models (DCTMs) have been applied in epidemiological studies frequently, especially in European studies [9, 55–58]. Some studies have incorporated spatially detailed modelling down to points of individual addresses [55, 56], while other models for PM_2.5_ were at a larger spatial scale of 1 km^2^ or above [9, 59]. DCTMs are deterministic models, using physical and chemical knowledge to model the dispersion and chemical transformations of emitted pollutants from sources. Compared to land use regression models, more effort and expertise are needed to collect input data. The quality of the input data is a key determinant of the performance of a DCTM. A wide variety of models exist that differ in the spatial scale which they cover (e.g. street, urban, regional, continental or even global scale) and the processes that they include (only dispersion versus dispersion plus chemical transport). A detailed discussion of DCTMs is beyond the scope of this review. A discussion of DCTMs focusing on particles is provided in various reviews [60–64].

Significant progress has been made in the application of DCTMs to the estimation of particle number concentration [65]. The uncertainty of emission factors is substantially larger than for fine particles. In the California Teachers study, a chemical transport model at the 4*4 km scale across California was applied to assess health effects of long-term exposure to chemical components in ultrafine and fine particles [66].

Validation with monitoring data is an important requirement for application of a DCTM. For long-term exposure studies, validation implies a spatial comparison of average modelling and monitoring data, which is more difficult to achieve than temporal comparison at one of a few sites. Few comparisons have been made between dispersion modelling and LUR modelling. A study comparing dispersion modelling and LUR in multiple European cities showed generally good agreement for NO_2_ [67]. Fewer dispersion models were available for PM, and the agreement with LUR models was less [67]. The size of respiratory health effects estimated in a birth cohort recruited from a large part of the Netherlands by a national LUR model and a detailed dispersion model was virtually identical for all pollutants [68].

### Satellite Remote Sensing

In the past decade, the use of satellite observations for assessing air pollution exposure in epidemiological studies has increased substantially [10, 69–71]. Satellite monitoring of aerosol optical depth (AOD) has contributed significantly to the development of global models of annual average PM_2.5_ concentrations used in the Global Burden of Disease (GBD) assessments [72, 73]. Useful satellite observations include especially nitrogen dioxide (NO_2_) and AOD, measured by the ozone-monitoring instrument (OMI) on board the Aura satellite and the moderate resolution imaging spectroradiometer (MODIS) and MISR instruments on board the Terra satellite, respectively [73, 74]. Monitoring methods are based on absorption and scattering of specific wavelengths of sunlight. These satellites pass each location of the earth at the same local time (~ 1330 hours for the Aura satellite and 1030 hours for the Terra satellite), allowing a consistent comparison across space. The instruments measure concentrations in the total atmospheric column, which are then converted to surface concentrations typically using the global chemical transport model GEOS-Chem [74]. Satellite observations of ozone are less useful, because of the high concentrations in the stratosphere, limiting the reliability of assessing surface concentrations from total column concentrations. The data are publicly available but require significant processing to be useful for human exposure assessment.

Several studies have documented that satellite observations correlate moderately well with surface measurements of NO_2_ and fine particles [72–75], particularly if the comparison is limited to background measurement locations. Globally, the spatial resolution of the NO_2_ and PM_2.5_ data is about 0.1° × 0.1°, translating to approximately 10 × 10 km. For North America, PM_2.5_ maps at 1 × 1 km have been developed, allowing assessment of intra-urban variations in exposure [76•]. For NO_2_, this is not possible yet. Because of the spatial scale of the observations, satellite data cannot represent the fine-scale variation related to, e.g. local traffic emissions. Studies in North America and Europe have suggested that satellite observations of air pollutants are especially useful in providing the (regional) background component of ambient air pollution in land use regression or other models [23, 77, 78]. The major advantage of satellite observations is that data are available globally, in contrast to surface-monitoring data that are available in a more limited number of countries and often concentrated in urban areas in those countries with sufficient monitoring. In international studies such as the global study on asthma [69] and the GBD assessment [79], the consistency of the measurement methodology is another advantage of satellite data. Concentration contrasts between countries derived from surface monitoring may be affected by differences in monitoring methods and selection of monitoring sites. Limitations of the satellite method include the temporally and spatially varying relationship between column and surface concentrations, the spatial resolution, interference by clouds and the characterization of a single time of the day [80]. Moreover, AOD does not give information about size distribution and chemical composition of the particles.

Several methods have been developed to overcome the problem of the temporally and spatially varying relationship between column and surface concentrations. In a series of North American studies, daily satellite AOD data were transformed into daily surface concentrations combining daily satellite observations, land use and surface monitoring of PM_2.5_ [81, 82]. Mixed effect models were used, allowing the relationship between column AOD and surface PM_2.5_ to vary from day to day and in space. Mixed models substantially improved the prediction of surface concentrations compared to an assumed constant conversion [81]. This novel methodology has now been applied outside North America, including Israel and Europe [80]. The methodology typically provides daily pollution estimates for a 1*1 km scale and allows spatially varying temporal daily exposures in time series studies of health effects of short-term air pollution exposures. After averaging, the method may be useful for long-term exposure studies as well.

A different approach was taken by van Donkelaar and co-workers to account for the spatially varying relationship between column and surface PM_2.5_ in North America and globally [76, 83]. The method starts to transform column AOD measurements into surface PM_2.5_ using the vertical profiles of the GEOS-Chem. The method thus uses geo-physical principles in contrast to the previously discussed method [81, 82] that translates AOD directly into surface PM_2.5_ using surface-monitoring data. The advantage is that the geo-physical method can be applied globally and in the absence of surface-monitoring data. Next, for North America, surface-monitoring data and land use information are used to reduce potential bias in the geo-physical-based surface PM_2.5_. The difference between direct satellite-transformed and surface-measured PM_2.5_ was regressed on various land use variables and particle composition [76]. Altitude and urban emissions were important factors resulting in difference between surface-measured and modelled PM_2.5_. Geographically weighted regression was used to develop the models, allowing the regression slopes to differ spatially.

A comparison between the application of satellite data with and without the use of additional surface monitoring and land use data for exposure assessment in a cohort study was conducted by Jerrett and co-workers [84•]. Seven different methods were applied to the baseline address of the ACS study, including satellite AOD only, satellite AOD adjusted to surface PM_2.5_ using geographically weighted regression and Bayesian methods further incorporating land use and traffic information. They reported that the relative risks of PM_2.5_ for circulatory and ischemic heart disease mortality in the ACS study were lower when exposure was estimated exclusively from satellites compared to ground-based estimates, supporting the use of additional data in exposure assessment using satellite remote sensing [84•].

## Combination of Methods

Recognizing the limitations of any single method, studies have developed hybrid models incorporating multiple methods in one framework. LUR models may be the method of choice if there is significant uncertainty about emission factors or physical-chemical transformation processes. LUR models cannot readily characterize atmospheric formation processes and because of their empirical nature are less transferable to other areas. Surface-monitoring data are typically spatially sparse, whereas models and satellite data are spatially more complete at the expense of more uncertainty of the concentration. Table 2 lists selected examples of hybrid models.

**Table 2:** Hybrid models for assessment of long-term air pollution exposure

Reference	Setting	Data combined	Combination framework
[85]	USA-scale LUR model for monthly average PM_2.5_	Surface monitoring, SAT, land use, traffic	SAT added as predictors in LUR identified with machine learning; Bayesian maximum entropy interpolation of residuals of the LUR model
[24•]	European-scale LUR model for annual average PM_2.5_ and NO_2_	Surface monitoring, CTM, SAT, land use, traffic	SAT and CTM added as predictors in LUR identified with supervised stepwise selection
[86]	USA-scale model for daily average PM_2.5_ at 1*1 km scale	Surface monitoring, SAT, CTM, land use, traffic, meteorology	Neural network to identify model, allowing non-linear and interaction effects
[26•]	Models for 14-day average concentrations of S, EC, Si and OC for six cities in the USA	Surface routine monitors, dedicated 14-day average monitoring,land use and traffic	Spatiotemporal model including lasso-based LUR model development and universal kriging
[91]	Daily average concentrations of 12 pollutants including NO_2_, O_3_, PM_2.5_ and PM_2.5_ components in Georgia state	Surface monitors and the CTM CMAQ	Data fusion method including kriging interpolation and regression

*CTM* chemical transport model, *SAT* satellite remote sensing

### Satellite Data in an Empirical Modelling Framework

Methods based upon satellite remote sensing data typically include other data to improve the assessment of surface PM_2.5_ and NO_2_ concentrations, as discussed in the “Overview of Methods” section. Reasons to include other data are to reduce bias in estimated surface PM and to improve spatial resolution, e.g. by including fine-scale traffic predictors. Novotny and co-workers reported that satellite NO_2_ was an important predictor in a national LUR model for the contiguous USA [78]. The model includes fine-scale traffic predictors as well. Beckerman and co-workers documented that remote sensing PM_2.5_ was a strong predictor of surface PM_2.5_ across the USA and substantially improved a LUR model further including land use and traffic data [85]. After Bayesian maximum entropy interpolation of residuals from the LUR model, little difference was seen between models with and without remote sensing PM_2.5_. The model without remote sensing PM_2.5_ exhibits more fine-scale variation [85]. De Hoogh and co-workers documented that the use of satellite AOD data and a large-scale chemical transport model (CTM) significantly improved European-scale LUR models for annual average PM_2.5_ concentrations developed from surface-monitoring data and land use and traffic data [24•]. NO_2_ LUR models were improved by the inclusion of CTM, with no additional improvement by OMI satellite data, related to the high correlation between OMI and CTM data.

Di and co-workers have used neural network methods to combine satellite AOD, land use, surface monitoring, CTM and a variety of different input data sources to develop a spatiotemporal model for the USA for PM_2.5_ [86]. A neural network was used to allow complex non-linear and interaction effects. On a global scale, Bayesian hierarchical models were used to derive annual average surface PM_2.5_ concentrations from satellite AOD, a global chemical transport model and surface-monitoring data [79, 87].

### Models Without Satellite Data

A series of papers from the University of Washington MESA-Air and NPACT study reported on spatiotemporal models incorporating spatial and temporal predictors in one model [27, 40, 88, 89]. Models were also developed and applied for particle composition [26]. The spatiotemporal model assesses 14-day average PM_2.5_ concentrations as a function of three features: spatially varying long-term means, spatially varying temporal trends and spatially varying and temporally-independent spatiotemporal residuals [26•]. The models include fixed daily surface monitor data at a limited number of sites, dedicated 14-day average spatially more resolved monitoring, land use and traffic.

A study in Catalonia used the Bayesian maximum entropy method to integrate NO_2_-monitoring data, LUR and CTM [90]. The Bayesian maximum entropy model performed better than the individual models.

Friberg and co-workers developed a data fusion method to model daily pollution estimates across Georgia [91]. The method combines daily model calculations by the chemical transport model CMAQ on a 12*12 km grid scale and daily monitoring data from routine monitoring stations for 12 pollutants including PM_2.5_, gaseous pollutants and five PM components. Data fusion allows the use of the spatial completeness of the CMAQ model and the temporal richness of the monitoring data.

## Selected Issues With Current Methods

### Historical Exposure Estimation

**Fig. 1:**
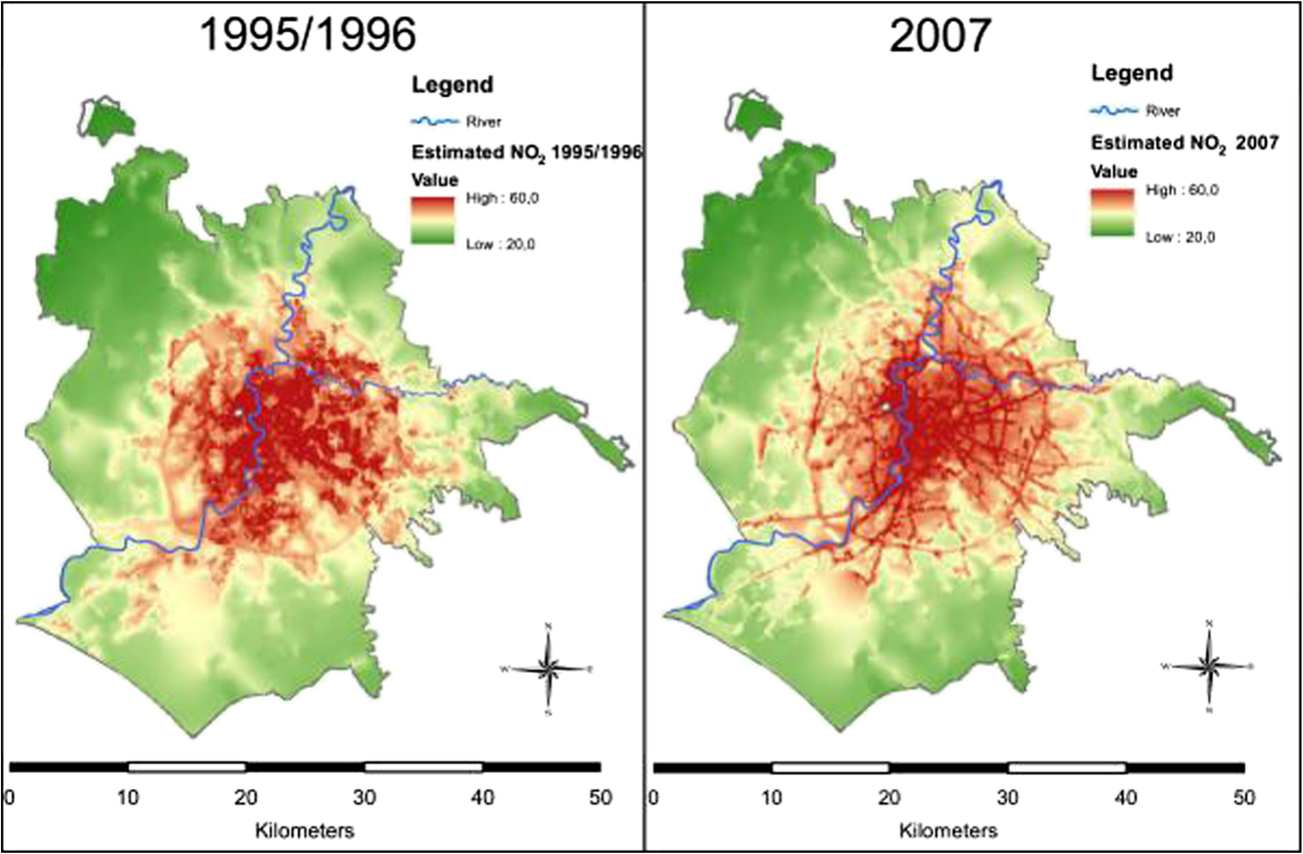
Map of modelled NO_2_ concentrations from the LUR model in Rome. (From: [94]; this work is licensed under a Creative Commons Attribution 2.0 Generic License)

An important issue in long-term air pollution exposure assessment is that the year in which measurements are conducted and/or the models are developed may be after recruitment of the study participants has taken place. An example is the large multicenter ESCAPE study where monitoring occurred mostly in 2009/2010 and recruitment went back to the mid-1990s in some cohorts [92]. In a Canadian national cohort, satellite data were only available after recruitment [10, 15]. A necessary assumption for application of recently developed models for long-term exposure that goes back in time several years is the spatial stability of air pollution contrasts. Studies in the Netherlands [93], Rome [94], Vancouver [95] and the UK [96] have shown that for periods up to 10 years, spatial air pollution contrasts of NO_2_ often remained stable. Figure 1 illustrates similar spatial patterns for Rome. LUR models based on current NO_2_ data may therefore predict intra-urban contrasts in exposure in the past well.

It is less certain whether this assumption of spatial stability over time applies to other pollutants, even though it is plausible that for other traffic-related pollutants including UFP, spatial stability can be expected. A study in Amsterdam, the Netherlands, documented that a LUR model developed in 2013 explained 36% of the variability of UFP measurements at 48 sites across Amsterdam 10 years earlier, though the absolute levels were underestimated [48]. The moderate explained variance could be due to either limited model performance or changes in spatial contrast. If the change in spatial contrast for UFP is larger than for NO_2_, more exposure estimation error occurs. In two pollutant models, the pollutant with the lowest exposure estimation error may remain most significant [16].

The stability of spatial air pollution patterns will not apply in rapidly developing areas. In a cohort study in China, the ranking of pollution of study areas changed during follow-up from 1998 to 2009 [97]. Methods to assess historic exposures related to changes in air pollution and residential address are discussed in [23, 98].

### Residence-Based Exposure Estimation and Personal Activity Patterns

Interpolation, dispersion and land use regression models provide individual estimates of exposure usually at a residential address, because of lack of availability of data of where people spend their time in cohort studies not designed to assess air pollution health effects. Time activity patterns such as the fraction of time spent at home, at work or at school and mode of and time spent in transport may be important determinants of personal exposure [99, 100]. Two studies evaluated the potential bias in air pollution epidemiology studies when exposure was characterized only at the residential address compared to also including the work/school address and commuting [101, 102]. Both studies reported only a modest bias towards the null when NO_2_ exposure was based upon the residential address only: ~ 15% lower effect estimates in a study in Basel [102], 16% lower effect estimates in Vancouver and 30% lower estimate in southern California [101]. This indicates that by improving exposure assessment, less biased risk estimates may be obtained [16]. The studies further suggest that bias may not be severe in studies accounting for a residential address only. The methodology to combine time activity patterns assessed by GPS or smartphone and fine-scale spatial maps is available [99], but currently cannot easily be applied to large populations [22].

### Vertical Gradients

Most models have largely ignored vertical gradients of air pollution, even if information is available in most CTMs. Geographical coordinates define the position using *x*- and *y*-coordinates but often do not have the height attached. In high-rise apartment buildings along major roads, this may be an important issue, as several monitoring studies have suggested important differences in air pollution related to height for homes located in major streets [103, 104]. This suggests that applying air pollution models in study areas where a large fraction of the population lives in high-rise apartment buildings, such as Hong Kong, may misclassify exposure. A study from Taiwan showed that the floor of the building was a significant predictor in a LUR model developed based upon monitoring at sites at low and high floors across the city [105].

## Conclusions

Methods to assess long-term exposure to outdoor air pollution have improved significantly over the past decade. New data and study designs have been applied, including satellite measurements of fine particles and NO_2_ and mobile monitoring designs to develop models for long-term UFP exposure. The methods applying satellite data for epidemiological studies are still improving rapidly, but have already proven to contribute significantly to national-, continental- and global-scale models. Spatiotemporal models have been developed allowing more detailed temporal resolution compared to spatial models typically for the annual average. The development of hybrid models including dispersion and chemistry transport models, satellite observations of NO_2_ and fine particles, land use and surface monitoring has improved models substantially. The use of new technology including GPS, smartphones and smaller pollution sensors may offer new possibilities to assess more individualized exposure.
